# Using Growth and Transpiration Phenotyping Under Controlled Conditions to Select Water Efficient Banana Genotypes

**DOI:** 10.3389/fpls.2019.00352

**Published:** 2019-03-26

**Authors:** Jelle van Wesemael, Ewaut Kissel, David Eyland, Tracy Lawson, Rony Swennen, Sebastien Carpentier

**Affiliations:** ^1^Laboratory of Tropical Crop Improvement, Division of Crop Biotechnics, KU Leuven, Leuven, Belgium; ^2^School of Biological Sciences, University of Essex, Colchester, United Kingdom; ^3^Banana Genetic Resources, Bioversity International, Leuven, Belgium; ^4^Banana Breeding, International Institute of Tropical Agriculture, Arusha, Tanzania

**Keywords:** biodiversity, banana, water use efficiency, real-time phenotyping, transpiration behavior, climate smart agriculture

## Abstract

Water deficit is one of the world’s major constraints in agriculture and will aggravate in the future. Banana (*Musa* spp.) is an important crop that needs vast amounts of water for optimal production. The International Transit Center of Bioversity International holds the world’s biggest collection of banana biodiversity (>1,500 accessions). The long-term aim of this research is to evaluate the potential within this collection for climate smart agricultural usage. Therefore, we developed a phenotyping setup under controlled environmental conditions and we selected 32 representatives of the *Musa* biodiversity (29 cultivars and 3 wild relatives) for evaluation. The best performing genotypes accumulated six to seven times more biomass than the least performing. Eight genotypes (five ABB, one AAB, and two AAA) invest under osmotic stress significantly more in root growth than in leaf growth. We predict therefore that these genotypes have potential for high productivity under rain fed conditions with a short dry season. To gain more insight in the transpiration physiology, we gravimetrically monitored individual plant transpiration over the diurnal period. All analyzed genotypes showed a marked reduction in transpiration rate in the afternoon. Moreover, the timing of this onset, as well as its impact on total transpiration, was genotype dependent. This phenomenon was more pronounced in 13 genotypes (eight ABB, two AAB, two AA, one BB). Banana is a crop originating from the humid tropics and has developed a strong root pressure to maintain an efficient water and nutrient transport even under saturated relative humidity conditions. Therefore, we hypothesize that the diurnal transpiration decline contributes to a higher water use efficiency without compromising the nutrient transport. Of the eight genotypes that had the best growth under osmotic stress, all analyzed ABB cultivars have a lower maximal transpiration rate, keep this maximal transpiration for a shorter time and therefore consume less water per day. We conclude that lab models are very useful to study the biodiversity and to identify different traits that contribute to a better drought tolerance/avoidance. We encourage researchers investigating other crops to start exploring their collections.

## Introduction

Agriculture is challenged to double food, feed, fiber, and fuel production in the 21st century (Foley et al., 2011). This requires sustainable intensification: increased agricultural production with sustainable use of natural resources and taking into account (future) climate variability. Improvement in crop management and cultivation can alleviate yield gaps, however, production remains subject to the inherent capacities of the used cultivars (Godfray et al., 2010; Negin and Moshelion, 2017). Available genetic diversity in wild relatives and/or (landrace) cultivars should be addressed to increase the adaptive character of (future) agricultural production systems while reducing the risks (Lipper et al., 2014; Dempewolf et al., 2017).

Banana (*Musa* spp.) is among the top 10 staple crops worldwide, with annual production of over 148 million tons (2016, FAOStat). The available diversity is substantial, however, an estimated 40% of the banana production, including the entire global export production, relies on a very narrow genetic group: the Cavendish banana (AAA genome) (Lescot, 2014). In general, banana requires vast amounts of water: between 1,200 and 2,600 mm/year, depending on the agro-ecological zone (Hegde and Srinivas, 1989; van Asten et al., 2011). Although cultivated in the humid (sub-)tropics, many production sites are vulnerable to seasonal drought, which impacts production severely. For example in the mild climate of the East African highlands, banana production requires 1,200–1,300 mm/year, but suffers a 8% yield decline per 100 mm water unavailable for evapotranspiration, making drought a major yield loss factor (van Asten et al., 2011).

In total over 1,500 banana accessions have been collected and stored in a germplasm collection, the International Transit Center (ITC, Bioversity International), hosted at the KU Leuven, Belgium. The banana diversity originates from various intra- and interspecific crosses between one or more ancestors (De Langhe et al., 2010). There are two common wild donors: *Musa acuminata*, the A genome donor, originating from the region Malaysia, Indonesia and Papua New Guinea, and *Musa balbisiana*, the B genome donor, originating from the region India, Myanmar, Thailand, and the Philippines (Perrier et al., 2011). Following hybridization and domestication, further reproduction has been vegetative and the genetic diversity was only enhanced through mutations. Most modern, edible cultivars are parthenocarpic, triploid hybrids (2n = 3x = 33x), classified based on their genomic constitution (AAA, AAB, or ABB). The presence of the B genome is reported to be beneficial for drought tolerance, attributed to its more northern, harsher, center of origin (Ekanayake et al., 1994; Thomas et al., 1998; Vanhove et al., 2012; Kissel et al., 2015; Janssens et al., 2016). Accessions carrying one or more B genomes have unique B specific alleles putatively contributing to drought tolerance (van Wesemael et al., 2018).

The phenology of banana is a function of its vegetative growth, determined by cultivar/subgroup specific thresholds (Taulya et al., 2014). Drought hampers growth by reducing cell expansion in roots, leaves, and fruits (Turner et al., 2007). During the vegetative growth stage, drought postpones bunch initiation and thus lowers the annual yield (Taulya et al., 2014). Even minor reductions in soil water content affect stomatal conductance and hence transpiration and photosynthesis (Turner et al., 2007). Banana plants tend to avoid leaf water potential falling below a critical water potential, and keep the leaves hydrated.

Worldwide only a few conventional banana breeding programs and efforts are successful and release improved varieties (Bakry et al., 1993; Ortiz and Vuylsteke, 1996; Creste et al., 2004; Ortiz and Swennen, 2014; Kubiriba et al., 2016; Brown et al., 2017; Dale et al., 2017). Currently the genetic parental base used for breeding efforts is small and requires expansion. Therefore, it is essential to identify more sources of resilience in adapted landrace cultivars for direct use, or alleles from crop wild relatives for breeding (Mickelbart et al., 2015). Germplasm collections (such as the ITC for banana) provide a valuable resource for such endeavors. To improve the turnover of the collection, its impact on cultivar selection/recommendations, and its input in breeding/cultivation programs, molecular and phenotypic characterization of such diversity is of utmost importance (Roux et al., 2011; Dempewolf et al., 2017). Molecular and cytological characterization is currently based on SSR markers, enabling the broad taxonomic description of the biodiversity (Christelová et al., 2016). Complementing these molecular efforts with functional phenotyping under controlled conditions provides valuable insights on growth patterns and allows a detailed study of different sub-traits contributing to drought tolerance/avoidance. These lab models allow rapid and objective screening of the vegetative growth potential under controlled, reproducible conditions relevant for a targeted agro-ecological environment. Evidently, the output from these lab models must be validated in the field (Vanhove et al., 2012).

The work presented here adds value to the banana germplasm collection by characterizing the growth potential of 32 genotypes under water limiting conditions. Firstly, we focused on identifying genotypes that grow vigorously under control conditions *and* show limited growth reduction under osmotic stress conditions. Secondly we studied the diurnal transpiration behavior in detail, seeking for a high plant production rate with a high water use efficiency.

## Materials and Methods

### Plant Material

*In vitro* banana plants were obtained from the International *Musa* Transit Center (ITC, Bioversity International, hosted at KU Leuven). A set of 32 genotypes, belonging to 23 triploid subgroups, and 3 additional diploid subspecies were selected based on morphological and taxonomic descriptions. This subset represents 573 genotypes of the *Musa* biodiversity (Table 1). Taxonomic categorization of the subgroups was derived from the *Musa* Germplasm Information System (MGIS, for ITC^1^, accessed October 11, 2018) and is based on Christelová et al. (2016). The 32 genotypes were arranged in 11 successive experiments. In each the reference cultivar (Cachaco, ABB, Bluggoe subgroup, ITC0643) was taken along with three other genotypes. Firstly, plants were phenotyped for performance under control and osmotic stress conditions in a growth characterization experiment. Secondly, only under control conditions, transpiration dynamics characteristics were assessed in a multi-lysimeter setup. Additionally a leaf level gas exchange experiment, measuring CO_2_ assimilation (*A*) and stomatal conductance to water (*g_s_*), was performed on the reference cultivar.

**Table 1:** Twenty-six subgroups, represented by 32 genotypes, spanning the *Musa* biodiversity, characterized by growth characterization (Gc) and transpiration dynamic (Td) experiments (x).

Subgroup	Genome	# Accessions in ITC	Taxonomic group	Representative genotype	ITC code	Gc	Td
Banksii	AA	/	42	Banksii	ITC0623	X	X
Zebrina	AA	/	34	Zebrina	ITC1177	X	X
Ambon	AAA	/	25	Pisang Bakar	ITC1064	X	X
Cavendish	AAA	51	26	Grande Naine	ITC0180	X	X
				Poyo	ITC0345	X	X
				Williams	ITC0365	X	X
Gros Michel	AAA	9	28	Gros Michel	ITC1122	X	X
Ibota	AAA	7	7	Khai Thong Ruang	ITC0662	X	X
Mutika/Lujugira	AAA	76	32	Mbwazirume	ITC1356	X	X
Red	AAA	10	24	Red Dacca	ITC0575	X	X
Rio	AAA	3	25	Leite	ITC0277	X	X
Unknown	AAA	/	/	Pisang Berangan	ITC1287	X	
Iholena	AAB	2	47	Uzakan	ITC0825	X	
Mysore	AAB	10	15	Pisang Ceylan	ITC1441	X	
Pisang Kelat	AAB	5		Pisang Palembang	ITC0450	X	X
Pisang Raja	AAB	4	40	Pisang Raja Bulu	ITC0843	X	X
				Pisang Rajah	ITC0587	X	X
Plantain	AAB	292	48	Orishele	ITC1325	X	
Pome	AAB	25	20	Foconah	ITC0649	X	X
				Prata Ana	ITC0962	X	X
Silk	AAB	14	19	Figue Pomme Géante	ITC0769	X	X
Bluggoe	ABB	16	45	Cachaco ^∗^	ITC0643	X	X
				Dole	ITC0767	X	X
Kluai tiparot	ABB	3	13	Kluai Tiparot	ITC0652	X	X
Monthan	ABB	10	45	Monthan	ITC1483	X	X
Ney Mannan	ABB	7	45	Blue Java	ITC0361	X	
Pelipita	ABB	4	/	Pelipita	ITC0472	X	
Peyan	ABB	2	/	Simili Radjah	ITC0123	X	X
Pisang Awak	ABB	17	16	Fougamou 1	ITC0101	X	X
				Namwa Khom	ITC0659	X	X
Saba	ABB	6	44	Saba	ITC1138	X	X
Pisang Klutuk Wulung	BB	/	14	Pisang Klutuk Wulung	ITC1587	X	X


*Eleven successive experiments were carried out, with each time Cachaco (^∗^) as reference cultivar. Per subgroup, the total number of accessions in the International *Musa* germplasm collection (ITC) is given as well as the corresponding taxonomic group (after Christelová et al., 2016).*

### Growth Characterization Experiment

#### Growth Conditions and Osmotic Stress Treatment

Throughout the experiments plants were grown in plant incubators (Aralab Fitoclima Bio 600, Portugal; Panasonic Sanyo, Japan; Bronson PGC-1400, Netherlands) with settings: light regime: 12 h/12 h (light/dark), temperature: 25°C, relative humidity: 75%. Plants were grown individually in containers with 350 mL medium: 361 mg/L KNO_3_, 121 mg/L K_2_SO_4_, 176 mg/L MgSO_4_.7H_2_O, 181 mg/L MgCl_2_.6H_2_O, 194 mg/L KH_2_PO_4_, 398 mg/L NaH_2_PO_4_.2H_2_O, 464 mg/L Ca(NO_3_)_2_.4H_2_O, 105 mg/L CaCl_2_.2H_2_O, 60 mg/L sequestrene, 1.1 mg/L H_3_BO_3_, 2.7 mg/L MnSO_4_.H_2_O, 0.23 mg/L ZnSO_4_.7H_2_O, 0.16 mg/L CuSO_4_.5H_2_O, 0.07 mg/L NaMoO_4_.2H_2_O, pH = 6 (modified from Swennen et al., 1986).

During 35 days prior to the growth characterization experiment, plants were adapted from the heterotrophic *in vitro* conditions to autotrophic conditions. After acclimation, the morphological characterization experiment lasted for 21 days. The stressed subgroup (*n* = 8) received a fresh nutrient solution including 5% polyethylene glycol (PEG-8000, Carl-Roth, Germany), an osmotic stress agent mimicking drought stress (-0.05 MPa, pF 2.7) (Michel, 1983). The control plants (*n* = 8) received the same nutrient solution without PEG-8000. We made sure that unwanted osmotic effects, caused by raised nutrient content, differential nutrient uptake and PEG concentration were limited, by frequent nutrient solution refreshment, according to age/consumption. This also avoided potential issues with lowered oxygen in the medium. No traces of contaminating heavy metal could be detected coming from the PEG.

#### Morphological Variables Extraction

At the start of the morphological characterization experiment the plants were phenotyped non-destructively (whole plant weight and pseudostem height), and the last formed leaf was marked. All plants were grown individually in sealed containers with only the above ground biomass (pseudostem and leaves) exposed to the surrounding environment. As such the depleted nutrient solution volumes were considered transpiration, and recorded at every refreshment.

After 21 days of growth, all plants were phenotyped again using combined (destructive) measurements and digital imagery. Firstly, the canopy area was calculated from top view images. Green plant pixels were separated from the blue background by color segmentation. Using a red reference surface of known size (10 × 5 cm) the green area was calculated. Secondly, the leaves were separated and spread out by increasing age and subsequently imaged. Based on these images the number of leaves, and individual leaf length, width, and area were determined. Image analysis was performed using an in house R tool based on the EBImage (Pau et al., 2010), and imagemagick packages. Pseudostem height, and fresh weights of root, pseudostem, and leaves were measured separately, and complemented by dry weight after 14 days of drying at 70°C. The final list of morphological variables (measured and calculated) can be found in Supplementary Table 1, 23 are taken up in the banana crop ontology database (Banana: CO_325^2^, accessed October 23, 2018).

An essential calculated variable is the above ground growth, the mass increase of the pseudostem and leaves. As the plants could not be destructively measured at the onset of the experiment, this was calculated based on allometric data from the final destructive phenotyping. We assumed that for non-stressed plants the proportion of shoot mass to the whole plant mass is stable over 21 experimental days. As such the initial above ground mass is the whole plant mass measured at the start of the experiment, multiplied by the cultivar specific proportion of above ground mass compared to whole plant mass.

The morphological characterization data of all 11 consecutive experiments was normalized toward the reference cultivar data (Cachaco, ABB) in order to reduce the experiment specific abnormalities (Eq. 1). For every variable (X) we calculated the median value per treatment for all reference plants over all experiments [median(X_allRef_Control_)] and per sub-experiment [median(X_subExpRef_Control_)]. The ratio of the median over all experiments to the median per experiment was used as a normalization factor. Subsequently this biological normalization factor (specific per sub-experiment and treatment) was applied to the other genotypes in that sub experiment and treatment.

(1) $$X_{\mathrm{norm}}=X_{\mathrm{notnorm}}\times \frac{\mathrm{median}(X_{\mathrm{all}\;\mathrm{Ref}})}{\mathrm{median}(X_{\mathrm{Sub}\;\mathrm{Exp}\;\mathrm{Ref}})}$$

#### The Effect of Osmotic Stress on Plant Performance

The plant performance (above ground biomass growth) in control and mild osmotic stress conditions was classified into five performance groups using k-means clustering based on the Euclidean distance. The above ground growth data were summarized (mean) per genotype for every treatment in order to group the genotypes. The k-means clustering used the ranks of growth per genotype in both conditions as input data. For two of these performance groups separately a sPLS-DA model (mixOmics, R-package, Cao et al., 2011) using five components, maximizes covariance between morphological variables (X) and the growth conditions (Y: 0% or 5% PEG). More precisely, using sPLS-DA, the original morphological variables are represented by artificial variables to investigate the correlation between the response (Y: 0% or 5% PEG) and those variables (phenotypic measurements). This allowed to rank the original variables based on the percentage change of the variable, taking into account the consistency of response over the different cultivars in the group. Finally, we selected, per performance group, the 10 most important (explanatory/changing) variables. To test whether the observed differences in performance group and treatment were significant a two way analysis of variance (ANOVA) was applied.

### Real-Time Transpiration Phenotyping

#### The Multi-Lysimeter Setup

Complementary to the morphological characterization we were interested in the within day transpiration patterns. To get a high resolution insight into the dynamics of transpiration throughout the day we set up a multi-lysimeter system. Plants were obtained through the ITC, and acclimated to autotrophic conditions for 35 days in a growth incubator with equal settings as above (acclimation of growth characterization experiment).

Plants of 35 days old were placed on 16–24 high precision balances (0.01 g accuracy, Kern, Germany) in a controlled climate chamber (light regime (Philips GreenPower LED): 12 h/12 h (light/dark), temperature 25°C, relative humidity 75%). These balances were connected to a computer and controlled by an in house developed Matlab tool, registering the weight of every plant every 10 s. Plants were grown in hydroponics solution (without PEG), in sealed 500 mL containers with only the above ground biomass open to the environment. As such the only weight loss approaches the transpiration through the plant system. After an adaptation phase of 2–4 days, data was collected for 6 consecutive days.

#### Transpiration Dynamics Variables Extraction

The registered weight data was converted to cumulative transpiration data by adding up differences between measurements. When light was switched on or off (start of the night, and start of the day, 12 h in between) data values were reset to zero. Per day segmented regression separated the day time (12 h) data into three segments by pinpointing two breakpoints (segmented, R-package, Muggeo, 2008). The night was added as one additional segment of 12 h whilst the light was switched off (no illumination). Finally, for every complete day (24 h) of phenotyping, 23 dynamic transpiration related variables were derived from the plant specific data based on the identified segments. The dynamic transpiration variables, measured every 24 h, were summarized (median) per plant (see Table 2 for all variables). Plant weight was registered before and after the trial. The daily acquired dynamic transpiration (rate) variables were normalized relative to the plant size on a daily basis. The plant size was estimated each day as a linear interpolation between the weight before and after the experiment.

**Table 2:** Twenty-three transpiration dynamic variables describing the daily transpiration pattern.

	Trait	Description	Unit	Normalized by plant mass	Type	Formula
A	duration_Start	Duration of the start segment	[h]		a	
B	duration_Maximum	Duration of the maximum transpiration segment	[h]		a	
C	duration_Reduction	Duration of the reduced transpiration segment	[h]		a	
D	relTransp_Night	Night time transpiration	[mL/g]	x	b	
E	relTransp_Start	Transpiration in the start segment	[mL/g]	x	b	
F	relTransp_Maximum	Transpiration in the maximum segment	[mL/g]	x	b	
G	relTransp_Reduction	Transpiration in the reduced segment	[mL/g]	x	b	
H	relTransp_Day	Day time (light on) transpiration	[mL/g]	x	b	E+F+G
I	relTransp_dayNight	24 h transpiration	[mL/g]	x	b	D+E+F+G
J	transp_Night_propDaily	Transpiration in the night relative to the 24 h transpiration			b	D/I
K	transp_Start_propDaily	Transpiration in the start segment relative to the 24 h transpiration			b	E/I
L	transp_Maximum_propDaily	Transpiration in the maximum segment relative to the 24 h transpiration			b	F/I
M	transp_Reduction_propDaily	Transpiration in the reduced segment relative to the 24 h transpiration			b	G/I
N	relTranspRate_Start	Transpiration rate in the start segment	[mL/(h^∗^g)]	x	c	E/A
O	relTranspRate_Maximum	Transpiration rate in the maximum segment	[mL/(h^∗^g)]	x	c	F/B
P	relTranspRate_Reduction	Transpiration rate in the reduced segment	[mL/(h^∗^g)]	x	c	G/C
Q	transpRate_Start_propNight	Transpiration rate in the start segment relative to the night time transpiration rate			c	N/(D/12 h)
R	transpRate_Maximum_propNight	Transpiration rate in the maximum segment relative to the night time transpiration rate			c	O/(D/12 h)
S	transpRate_Reduction_propNight	Transpiration rate in the reduced segment relative to the night time transpiration rate			c	P/(D/12 h)
T	transpRate_Day_propNight	Transpiration rate in the day relative to the night time transpiration rate			c	H/D
U	relmLSave	The volume of water saved by the reduction feature	[mL/g]	x	d	[E+F+(C ^∗^ O)]-H
V	closureProp	The relative decrease of transpiration rate between maximal and reduced segment			d	1-(P/O)
W	propSave	The volume of water saved by the reduction feature relative to the whole day transpiration			d	1-[H/(E+F+(C ^∗^ O))]


*Every variable is depicted by a letter, corresponding to Figure 3, formulas are derived by letters. Variables are classified (type column): (a) the duration of every segment, (b) the segment specific transpiration volume, (c) transpiration rates relative to the night water loss rate, (d) the relative decrease of transpiration rate between maximal and reduced.*

#### Phenotypic Similarity Based on the Transpiration Pattern

Genotypes are clustered based on phenotypic similarity within the daily transpiration profile. First, the data collected for 23 variables on multiple days was summarized (mean per genotype) and scaled. Secondly, blind hierarchical clustering was performed based on the average difference of measured variables between genotypes. This finally led to a similarity tree. sPLS-DA toward the separated clusters (X: 23 transpiration variables, Y: transpiration clusters) reveals the transpiration dynamics variables underlying the genotype similarity. ANOVA on the individual variables identified those which separated both transpiration phenotypes significantly. In order to avoid confounding the results with a multitude of reference cultivar data, the sPLS-DA and ANOVA performed only used data of one sub-experiment for the reference cultivar.

### Diurnal Gas Exchange Patterns

To validate the transpiration dynamic behavior of our reference cultivar, gas exchange analysis was performed at the leaf-level. CO_2_ assimilation (*A*) and stomatal conductance to water (*g_s_*) were measured every minute on the middle of the second youngest fully developed leaf using a LCpro-SD infrared gas analysis system (ADC BioScientific Limited, United Kingdom). Intrinsic water use efficiency (iWUE) was calculated as the ratio of *A* and *g_s_*. Plants were grown in 800 mL pots filled with peat-based compost (Levingtons F2S, United Kingdom) and placed in a controlled climate chamber [light regime: 12 h/12 h (light/dark), 350 μmol/m^2^/s, temperature 25°C/22°C (day/night), relative humidity 75%]. The leaf cuvette maintained identical conditions. Measurements were performed on five replicates.

### Data Processing and Availability

All variable extraction and data processing was carried out in R (V 3.4.3). Data is available through the Harvard-Dataverse repository (doi: 10.7910/DVN/NHVBN5).

## Results

### Growth Performance

#### Genotype Performance Ranking

**FIGURE 1:**
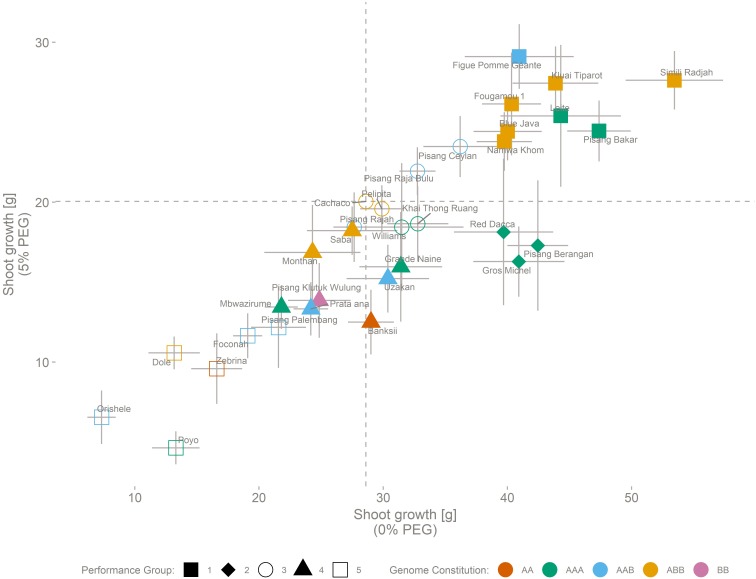
Growth performance of 32 genotypes representing the *Musa* biodiversity. Performance is based on the above ground biomass production, under control (0% PEG) conditions (horizontal axis) and stressed (5% PEG) conditions (vertical axis). SE (*n* = 8) is indicated for control and stress for each cultivar. The cultivar genomic constitution (AA, AAA, AAB, ABB, or BB) is depicted by colors, while shapes indicate the result of a 5 kern k-means clustering. Dashed lines indicate the growth of the reference cultivar (Cachaco) under control and stress conditions. Growth (0% PEG) = 3.70 + 1.53 ^∗^ Growth (5% PEG) (*n* = 32, *R*^2^_adj_ = 0.77).

The performance of 32 genotypes representative for the *Musa* biodiversity was assessed based on vegetative growth of above ground plant parts in control (0% PEG) and osmotic stress (5% PEG) conditions (Figure 1). Simili Radjah (ABB) was the best growing cultivar under control conditions, while Figue Pomme Géante (AAB) had the highest growth under osmotic stress. The best performing genotypes grew six to seven times better than the least performing genotype (stress-control, respectively) (Figure 1 and Table 3). The growth in control and stress conditions was correlated [Growth (0% PEG) = 3.70 + 1.53 ^∗^ Growth (5% PEG), *n* = 32, *R*^2^_adj_ = 0.77].

**Table 3:** Growth performance of 32 genotypes representing the *Musa* biodiversity.

Genotype	Genome	0% PEG	5% PEG	Growth inhibition (%)
Figue Pomme Géante	AAB	40.95 ± 4.38	29.11 ± 2.02	28.9^*^
Simili Radjah	ABB	53.45 ± 3.92	27.62 ± 1.83	48.3^***^
Kluai Tiparot	ABB	43.87 ± 3.45	27.44 ± 2.3	37.5^**^
Fougamou 1	ABB	40.34 ± 2.38	26.14 ± 3.21	35.2^**^
Leite	AAA	44.3 ± 4.85	25.4 ± 4.44	42.7^*^
Pisang Bakar	AAA	47.38 ± 2.56	24.46 ± 1.9	48.4^***^
Blue Java	ABB	40.02 ± 2.74	24.43 ± 1.81	39^∗∗∗^
Namwa Khom	ABB	39.75 ± 2.22	23.79 ± 1.83	40.1^***^
Pisang Ceylan	AAB	36.2 ± 2.96	23.48 ± 1.91	35.1^**^
Pisang Raja Bulu	AAB	32.76 ± 1.45	21.94 ± 1.49	33^∗∗∗^
Pelipita	ABB	29.9 ± 1.75	19.59 ± 1.48	34.5^***^
Khai Thong Ruang	AAA	32.78 ± 2.47	18.65 ± 2.36	43.1^**^
Pisang Rajah	AAB	27.67 ± 1.68	18.44 ± 2.18	33.3^**^
Williams	AAA	31.5 ± 4.97	18.44 ± 4	41.5^nS^
Saba	ABB	27.5 ± 3.64	18.22 ± 1.51	33.7^*^
Red Dacca	AAA	39.7 ± 3.99	18.14 ± 4.58	54.3^**^
Pisang Berangan	AAA	42.45 ± 2.44	17.29 ± 4.08	59.3^***^
Monthan	ABB	24.3 ± 3.87	16.86 ± 2.96	30.6^nS^
Gros Michel	AAA	40.92 ± 3.67	16.29 ± 2.2	60.2^***^
Grande Naine	AAA	31.41 ± 3.33	15.96 ± 3.43	49.2^**^
Uzakan	AAB	30.37 ± 3.32	15.22 ± 2.12	49.9^**^
Pisang Klutuk Wulung	BB	24.86 ± 2.52	13.85 ± 2.34	44.3^**^
Mbwazirume	AAA	21.8 ± 1.33	13.44 ± 1.36	38.3^***^
Prata Ana	AAB	24.18 ± 1.4	13.33 ± 1.66	44.9^***^
Banksii	AA	29.01 ± 1.85	12.5 ± 2.02	56.9^***^
Pisang Palembang	AAB	21.57 ± 2.21	12.17 ± 2.54	43.6^*^
Foconah	AAB	19.09 ± 1.17	11.65 ± 1.4	39^∗∗^
Dole	ABB	13.17 ± 2.07	10.58 ± 1.02	19.7^nS^
Zebrina	AA	16.6 ± 2.05	9.59 ± 2.2	42.2^*^
Orishele	AAB	7.32 ± 1.14	6.55 ± 1.67	10.5^nS^
Poyo	AAA	13.3 ± 1.91	4.64 ± 1.03	65.1^**^


*Mean aboveground biomass production (±SE) in 0% PEG and 5% PEG conditions (n = 8). Growth inhibition (%) calculated as the difference between mean aboveground biomass in 0% PEG and 5% PEG relative to the mean in 0% PEG. Significance of *t*-test (growth in 0% PEG compared to 5% PEG) indicated (^∗∗∗^*p* < 0.001, ^∗∗^*p* < 0.01, ^∗^*p* < 0.05, nS: *p* > 0.05).*

Based on k-means clustering, five distinct performance groups were identified capturing 92% of the total sum of squares (Figure 1). Group 1 (Figue Pomme Géante, Kluai Tiparot, Simili Radjah, Fougamou 1, Leite, Blue Java, Pisang Bakar, and Namwa Khom) and group 2 (Red Dacca, Pisang Berangan, and Gros Michel) were vigorous growers under control conditions. Two-way analysis of variance between the performance groups 1 and 2 and the treatment followed by a Tukey HSD test pointed out that plant growth was significantly different between these two performance groups under 5% PEG conditions while this was not the case under control conditions [5% PEG: group 1 – group 2: *p* = 0.0004, *n* = 64/24 (Group 1/Group 2); 0% PEG: group 1 – group 2: *p* = 0.59, *n* = 64/24 (Group 1/Group 2), Figure 1]. One group of genotypes (Poyo, Orishele, Dole, Zebrina, Foconah, and Pisang Palembang) grew poorly under control and osmotic stress conditions.

#### The Differential Impact of Osmotic Stress

**FIGURE 2:**
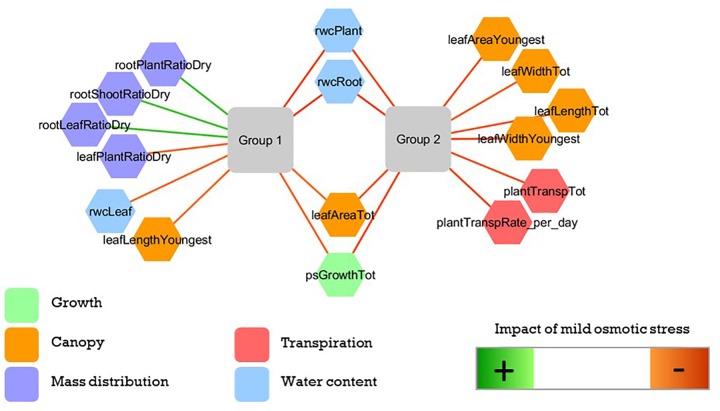
The variables most explanatory for the difference between stress and control derived independently in performance groups 1 and 2. Figure based on 11 cultivars from performance classes 1 and 2. Green connecting lines indicate that the variable is increased in mild osmotic stress compared to control, red lines indicate a decreased value.

To further investigate the effect of the osmotic stress treatment we examined data from performance groups 1 and 2, together representing 11 (out of 32) genotypes. In each group a sPLS-DA, maximizing covariance between 39 morphological variables (X) and the plant growth conditions (Y), separated plants grown in 0% PEG from those grown in 5% PEG using five sPLS-DA components. The correlation coefficient between the morphological variables matrix and the sPLS-DA model indicates which variables change most between plants grown in both conditions. In these performance group specific models the contribution of each morphological variable in explaining the plant growth condition was ranked. This enabled to select the most important variables per performance group (Figure 2). Only four of these variables refer to a mutual stress effect in both performance groups: all selected genotypes showed a reduction in total leaf area, the pseudostem growth and a change in the relative water content in the root and whole plant. Group 1 genotypes showed a larger mass redistribution in favor of the roots (Figure 2 and Supplementary Table 2). The latter was also observed when the below ground growth was assessed (Supplementary Figure 1). Univariate statistics (ANOVA) validated these multivariate findings (Table 4). All selected variables showed a significant impact of the osmotic stress treatment (ANOVA, *p* < 0.001, Table 4). Five variables showed a significant (*p* < 0.001) (treatment × performance group) interaction: rootPlantRatioDry, rootShootRatioDry, and rootLeafRatioDry seriously increased for group 1, while leafWidthTot and leafWidthYoungest seriously decreased in group 2 (Table 4 and Supplementary Table 3).

**Table 4:** ANOVA results showing the significance level of the treatment, performance group and their interaction effect for the morphological variables explaining most of the growth difference under both treatments.

Morphological variable	*P*-value		
	
			
	Treatment	Performance group	Treatment × performance group
rootPlantRatioDry	6.86E-24	1.13E-07	6.52E-05
rootShootRatioDry	4.25E-24	2.04E-07	5.72E-05
rootLeafRatioDry	1.05E-24	1.8E-06	0.000563
leafPlantRatioDry	1.08E-11	0.004	0.069
rwcLeaf	1.66E-16	0.038	0.238
leafLeafYoungest	5.2E-19	0.533	0.013
rwcPlant	7.5E-31	0.076	0.023
rwcRoot	1.02E-36	0.16	0.694
leafAreaTot	1.22E-19	0.047	0.02
psGrowthTot	1.32E-23	1.54E-05	0.103
leafAreaYoungest	5.56E-20	0.005	0.02
leafWidthTot	3.28E-10	0.043	0.000464
leafLengthTot	4.92E-17	0.064	0.009
leafWidthYoungest	1.1E-16	7.04E-06	0.000578
plantTranspTot	1.36E-16	3.75E-07	0.032
plantTranspRate_per day	1.35E-11	4.79E-09	0.089


*Two performance groups are used: group 1 (eight cultivars) and group 2 (three cultivars). Eight unique plants per treatment and cultivar.*

### Real-Time Transpiration Monitoring

#### The Daily Transpiration Pattern in a Non-changing Environment Is Not Constant

**FIGURE 3:**
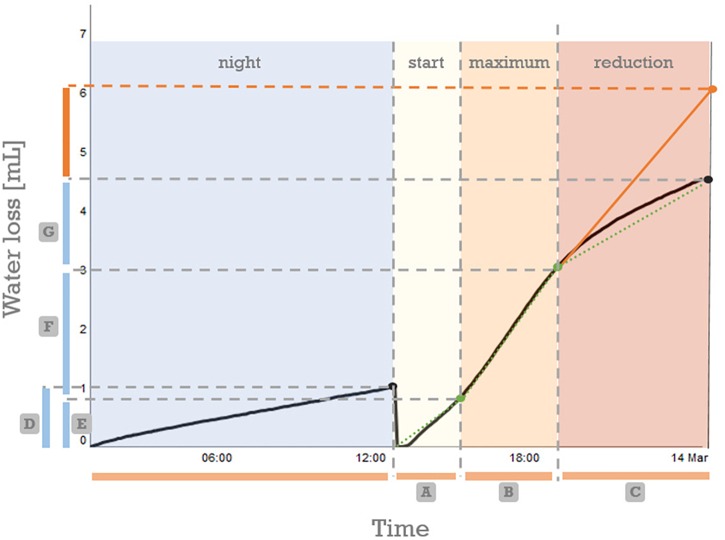
Segmented regression on the cumulative (daily) transpiration pattern allows to separate the transpiration into four distinct parts. For calculation purposes the data is reset to zero at every light switch (12:00 and 00:00). Based on the breakpoints (indicated green dots) derived by segmented regression analysis different base variables are directly derived from the graph: (A) duration of the start segment, (B) duration of the segment with maximal transpiration, (C) duration of the segment with pre-night reduced transpiration, (D) the volume of water lost during the night, (E) the water loss during the start segment, (F) the volume of water lost in the maximum segment, (G) the water lost in the reduced transpiration phase. For all 23 calculated variables using these based variables, see Table 2.

We investigated the transpiration behavior of 26 genotypes in more detail. The transpiration in a homogenous, non-changing environment was not constant (Figure 3 and Supplementary Figure 2). Every genotype showed a marked reduction in transpiration rate in the afternoon, however, the timing of this onset, as well as its impact on total transpiration was genotype dependent (Supplementary Figure 2).

Using segmented regression more insight in the sub daily transpiration pattern was observed. The daily (24 h) transpiration pattern could be split up into four parts: night, start, constant maximum, and reduction (Figure 3). Over each of these four segments the transpiration volume (mg ≈ mL) and segment duration (h) were determined. This allowed us to further describe the diurnal transpiration pattern using 23 variables (Supplementary Table 3). The variables could be grouped in four categories, according to their connotation: (a) describing the duration of the segments, (b) related to the segment specific transpiration volumes, (c) related to the segment specific transpiration rates, and (d) related to the impact of the last segment, the segment characterized by a pre-night transpiration rate reduction.

#### Two Transpiration Phenotypes Are Observed Within the Musa Biodiversity

**FIGURE 4:**
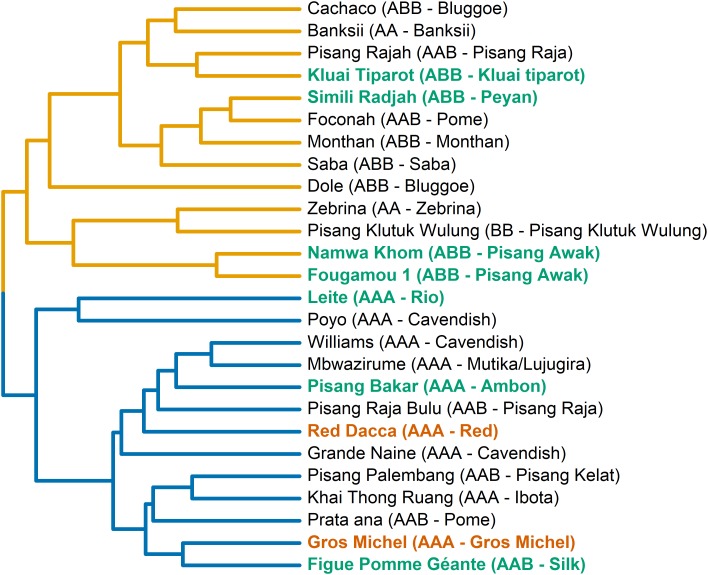
Two transpiration phenotypic groups are separated based on the phenotypic similarity tree. The tree is constructed based on the average phenotypic difference between genotypes. The transpiration phenotypic groups are indicated here by the color of the lines in the tree (yellow: group A, blue: group B). To couple to the growth performance (Figure 1) cultivars from performance group 1 are colored in green, and group 2 in red. Names of cultivars, their genomic constitution and the subgroup they belong to are given.

Based on those 23 variables, two transpiration phenotypes were blindly separated via hierarchical clustering (Figure 4). In general, genotypes representing the same, or similar, taxonomic subgroups clustered into the same group. For example all ABB cultivars cluster in group A while all AAA cultivars cluster in group B (Figure 4). Diploid genotypes cluster in group A irrespective their genome background.

**FIGURE 5:**
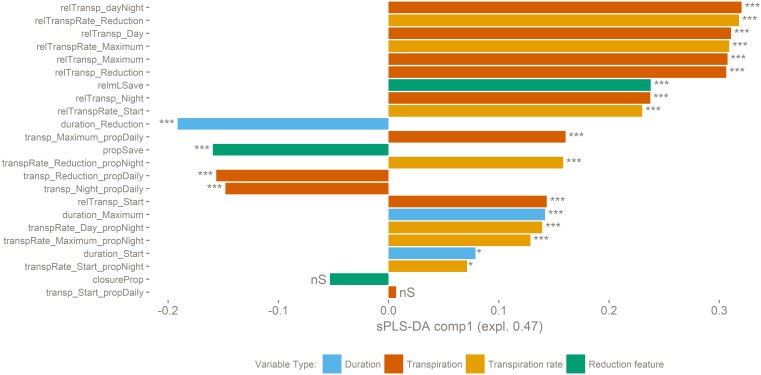
Transpiration dynamics related variables underlying the separation into both transpiration groups are ranked according to their sPLS-DA loadings score. A positive score on the *X*-axis is correlated to group B, a negative to group A. Variables with a significant difference between transpiration phenotypic groups indicated (*t*-test, ^∗∗∗^*p* < 0.001, ^∗^*p* > 0.05, nS: *p* > 0.1). The genotypes belonging to group B have a higher maximum transpiration rate and keep that high rate for a longer time. This makes that they have a higher total transpiration and that they save less water compared to genotypes of group A.

The importance of the 23 variables was calculated by sPLS-DA (Supplementary Figure 3). Nineteen out of 23 analyzed variables were significantly different (*P* < 0.001) between the two transpiration groups (Figure 5). Mean values of the variables per transpiration phenotype group are given in Supplementary Table 3.

**FIGURE 6:**
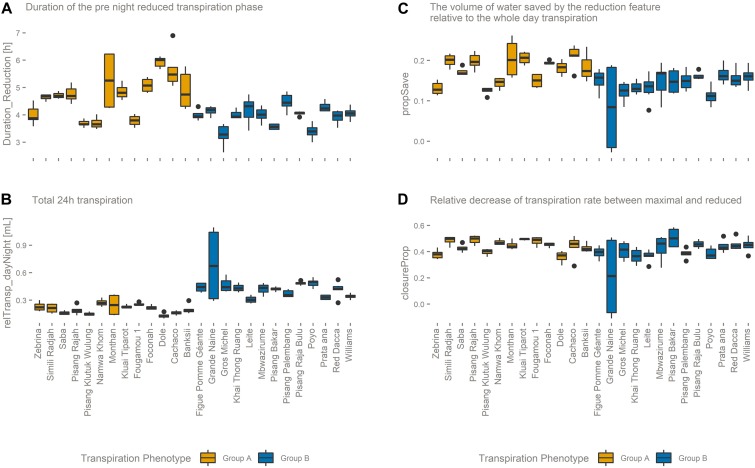
**(A)** The duration of the reduced transpiration segment (duration_Reduction) is longer for transpiration phenotype group A genotypes. **(B)** The total 24-h transpiration (relTransp_dayNight) is on average higher for transpiration phenotype group B genotypes. **(C)** The relative volume of water saved by the afternoon transpiration reduction (propSave) is higher in transpiration phenotype group A genotypes, although **(D)** both transpiration phenotypic groups manage the same reduction in transpiration rate (closureProp).

The genotypes belonging to group B have a higher maximum transpiration rate and keep that high rate for a longer time. This means that they have a higher total transpiration and that they save less water compared to genotypes of group A. In general, the transpiration phenotypic group B is positively correlated to variables related to water consumption. For example, Poyo (AAA) belonging to group B showed the highest average daily transpiration volumes per unit biomass, while the genotype transpiring the least, Dole (ABB) belonging to group A, on average transpired 4.5 times less water per unit of biomass (relTransp_dayNight, *p*-value < 0.001, Figure 6B). Furthermore, the opposite phenotypic group A is characterized by the higher importance of the reduced transpiration phase (Figure 6A and Supplementary Figure 2). For example, the duration of the reduced transpiration segment displays a 2.6 h difference between Dole (ABB) (Group A) and Gros Michel (AAA) (Group B) (duration_Reduction, *p*-value < 0.001, Figure 6A). There was no significant group segregation when looking at the pre-night closing potential (closureProp, *p*-value > 0.1, Figure 6D). This is the ratio of the maximal transpiration rate to the transpiration rate during the reduction phase, and both transpiration groups have the same closing potential. However, the timing of reduction onset cause that group A genotypes saved significantly more water in the reduction phase (propSave, *p*-value < 0.001, Figure 6C) relatively to group B cultivars. Some genotypes managed to save more than 20% of their daily (day time) water loss by reducing their transpiration rate by 45% (Figure 6C,D).

### Diurnal Leaf-Level Gas Exchange Patterns

**FIGURE 7:**
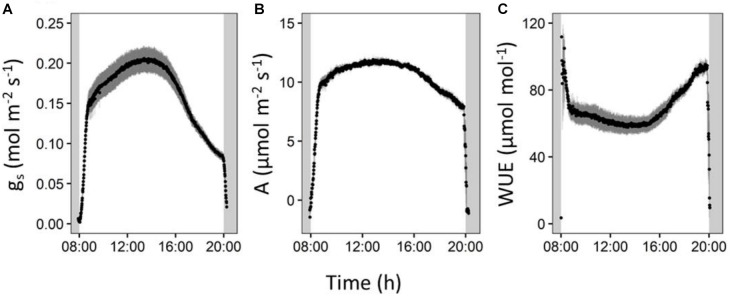
Reference cultivar (Cachaco, Bluggoe, ABB) leaf gas exchange measurements performed on the second youngest leaf. **(A)** Stomatal conductance shows a marked decrease in the afternoon. **(B)** The CO_2_ assimilation also shows an increase, but **(C)** the intrinsic water use efficiency rises in the afternoon.

The occurrence of an afternoon transpiration reduction was confirmed by diurnal gas exchange measurements. During the afternoon, stomatal conductance in our reference cultivar Cachaco (ABB) decreased by 59% (Figure 7). Simultaneously we observed a decrease in CO_2_ assimilation of 34% (Figure 7). However, the decrease in CO_2_ assimilation was small compared to the decrease in stomatal conductance, meaning that relatively more CO_2_ is taken up for each molecule of water transpired and represents a strong afternoon increase in intrinsic water use efficiency (iWUE) of 61% (Supplementary Figure 4).

## Discussion

### Risk Taking Under Osmotic Stress

The laboratory model presented here provides an objective genotype ranking under two relevant water potential treatments (Figure 1 and Table 3). For banana, biomass production is a good proxy for yield. Taulya et al. (2014) demonstrated an excellent correlation between the harvest index and total dry weight at harvest. Therefore, we focus the discussion on those genotypes that had a vigorous (vegetative) growth under control conditions. When plants are exposed to osmotic stress at the root level, a root derived ABA signal is triggered, leading to a decrease in stomatal conductance, and transpiration. This affects CO_2_ diffusion into the leaf and lowers the photosynthetic rate, decreasing overall growth. Under osmotic stress the allometric relations of genotypes belonging to performance group 1 change due to a relative root mass increase, more than a decrease in above ground mass (Figure 2, Table 4, and Supplementary Figure 1). This genotype specific reaction to lowered water availability has been observed in other *Musa* trials (Sebuwufu et al., 2004; Delfin et al., 2016). Investment in root biomass is a strategy which can lead to drought avoidance and supports further transpiration and growth, but holds a certain risk (Tardieu et al., 2017). It enables the exploration of a larger (soil) volume for water uptake, but it has a significant respiration cost, an investment that will be lost if no extra water is found (Tardieu et al., 2017). The success of this risky drought avoidance phenotype depends on the actual climate conditions throughout the growth period. During a relatively short dry season, followed by a rainy season with excess water supply, the deep rooting phenotype is favored as it largely avoids drought and supports continued transpiration and growth during the adverse conditions. This reduces the impact of stress on the yield compared to phenotypes showing growth cessation. In those environmental conditions, the cultivars of performance group 1 seem to be more suitable. Our laboratory model was designed to identify genotypes suitable for those conditions. However, under prolonged drought conditions the cost of root investment can be too high. Then a survival based strategy is preferred and a different lab model needs to be set up. In episodic, high frequency drought scenarios, rapid continuation of (root) growth upon rewetting could be preferred, but would also require a different lab model (Comas et al., 2013; Pierik and Testerink, 2014).

### A Closer Look at the Daily Transpiration Pattern and Its Putative Impact on Crop Productivity

Originating from the humid tropics, banana is adapted to growth in nearly saturated humidity conditions. The plant remains hydrated while transport of water and nutrients are ensured through a strong root pressure (Turner et al., 2007). During the photoperiod, banana, a C3 plant, manages stomatal aperture to balance water loss with carbon uptake, thereby avoiding dehydration and maintaining an appropriate photosynthetic rate. An iso/anisohydric classification of a plant or species depends on the definition used and the environment in which it is grown. The hydraulic parameters related to iso/anisohydric terminology are not only dependent on the genotype/species, but are also influenced by the environment; for example by the soil water potential, the soil-to-canopy-hydraulic conductance, the leaf water potential, and the maximal transpiration rate (Hochberg et al., 2018). When the evaporative demand exceeds the supply of water to the transpiration stream, stomata respond to prevent the leaf water potential from falling below a critical level. The main driver controlling this stomatal closure is ABA. This hormone can be sent by the roots when soil water potential is too low but can also be released by the leaves when the critical leaf water potential is reached. In our controlled conditions the transpiration pattern showed a decline toward the end of the daylight period without a change in environmental conditions (Figure 3 and Supplementary Figure 2). All genotypes reduce the transpiration rate by the end of the day by between 40 and 50% of the maximal transpiration rate (closureProp, Figure 6D). This phenomenon has already been described in bean and it has been shown that the stomatal responsiveness depends on the time of the day (Mencuccini et al., 2000). The authors hypothesized that a diurnal pattern is responsible for the changed stomatal responsiveness. We confirm this decline in transpiration in banana and moreover show that there is a genotypic dependence (Figure 4). Cultivars mainly differ in the timing of the transpiration reduction (duration_Reduction) and the transpiration rates distinguish two transpiration phenotypes (Figure 5, 6). Also for this trait we have identified risk takers. Genotypes belonging to group B reduce their transpiration relatively late. Genotypes belonging to group A behave more conservative and reduce their transpiration sooner. In our experiment, environmental factors, possibly causing a decrease in stomatal conductance, were absent. A diurnal factor as hypothesized by Mencuccini et al. (2000) that reduces the responsiveness of stomata toward the light induced opening impulse can lead to the observed phenotype. Stomatal aperture relies on a balance between responsiveness to signals to (re)open and the responsiveness to signals to (re)close. The latter is mainly under ABA control (Kollist et al., 2014), therefore the significant differences observed between the genotypes could be explained by a different sensitivity to the opening impulse but could also be a different sensitivity toward a closing impulse. So a difference in ABA sensitivity/production or a feedback to sugar sensing as suggested by Delorge et al. (2014) could also lead to the observed group differences. It is not clear why this mechanism of declining transpiration evolved. A reduction in transpiration that is not related to a change in irradiance, internal CO_2_, VPD or soil water potential does not seem to be desired in terms of plant productivity. However, the growth ranking of our genotypes (Figure 1) does not indicate any negative effect of such conservative transpiration behavior, as the cultivars in the preferred growth performance group (Group 1) do not exclusively belong to transpiration group A (Figure 1, 4). For example Figue Pomme Géante and Simili Radjah are both well performing cultivars, but they have distinct transpiration phenotypes.

The lower stomatal conductance during the afternoon restricts CO_2_ uptake for photosynthesis, resulting in a CO_2_ assimilation decrease (Figure 7). However, this CO_2_ assimilation decrease is moderate compared to the decrease in stomatal conductance, meaning that relatively more CO_2_ is taken up for each molecule of water transpired. This was represented by a strong afternoon increase in intrinsic water use efficiency (iWUE) (Supplementary Figure 4). The afternoon stomatal closure thus results in a relative water saving while maintaining the growth potential. Our results show that this reduced afternoon transpiration reduction is a promising (pre-)breeding screening trait as not all cultivars show this to the same extend, and it does not seem to compromise the growth since four of the eight investigated genotypes belonging to growing phenotype group 1 belong to transpiration phenotype group A (Figure 1, 4).

### The Osmotic Stress Mimicked in the LabIs Representative

Each agro-ecological environment has specific constraints and has its ideal niche cultivar. As such it is crucial to mimic those specific environmental conditions as closely as possible. We used 5% polyethylene glycol (PEG), a well-known osmotic agent, to subject plants to a constant lowered water availability of -0.05 MPa (pF 2.7) for 21 days. A water potential of -0.05 MPa is considered a mild drought stress (Michel, 1983; Verslues et al., 2006; Kissel et al., 2015). The growth in these conditions reduced to 58% of the control growth (Table 3). This is comparable to the effect of the mild stress treatment in the study of Kissel et al. (2015) where various banana cultivars were phenotyped in relevant (actual) conditions in pots in Uganda. Also plants in a field based experiment, grown for 63 days without irrigation, causing a significantly lower soil water content, showed reduced growth to 61% of the fully irrigated plants (Mahouachi, 2007). The relevance of a lab model relies on selecting appropriate treatment conditions (Negin and Moshelion, 2017). The stress effect in other experiments is thus similar to the effect in our lab model, strengthening the relevance for biodiversity screening purposes.

Many agro-eco zones where bananas are grown on a rain fed basis have a dry season of 1–3 months during the crop cycle of 10–14 months. So in these environments, ideal cultivars grow vigorously under non-restricting water conditions with a minimal setback during the dry season. The growth ranking in Figure 1 evaluates the genotype performance based on the above ground biomass, a yield proxy. As mentioned above, all genotypes are impacted by the osmotic stress, on average growth reduced to 58% of the control growth (34–89%, min-max) (Figure 1 and Table 3). The performance under control conditions was correlated to the performance under stress conditions (*R*^2^ = 0.77), implying that sturdy plants under control conditions are also more likely to perform better under stress. The most promising genotypes for that kind of environments have limited setback under 5% PEG (group 1) and have a water saving transpiration behavior (group A) [e.g., Fougamou, Namwa Khom, Simili Radjah, and Kluai Tiparot (all ABBs)]. The cultivars belonging to group 2 [Pisang Berangan, Gros Michel, and Red Dacca (all AAAs)] had a significantly impacted growth due to the reduced water availability and all investigated genotypes belonged to group B for their transpiration behavior. This makes them less suitable for agro ecological zones with an expected dry period. Drought during the vegetative stage causes a growth delay, postponing bunch initiation and flowering, thus reducing the harvest (Taulya et al., 2014). The results presented here indicate that many, but not exclusively, genotypes with a B genome contribution perform better under water limiting conditions. All the investigated ABB genotypes (eight in total, Figure 4) belonged to group A meaning that they had a lower maximum transpiration rate and kept that high rate for a shorter time.

## Conclusion

Drought is a complex phenomenon and tolerance/avoidance is achieved via multiple sub-traits that are not necessarily correlated. We designed two lab models to investigate important sub-traits that contribute toward drought tolerance/avoidance and we presented the variables that were most important to quantify those traits. We conclude that banana has a diurnal transpiration pattern in which stomatal conductance decreases near the end of the day. We present the diversity in transpiration patterns and observe that there are genotypes with a more conservative water saving behavior (group A) which increases the water use efficiency. We classify eight genotypes that have a good potential to have a high production under a rain fed growing system with a short dry season (group 1). These genotypes continue to grow under mild osmotic stress and invest under mild drought stress more in root growth than leaf growth. Four of the eight investigated genotypes belonging to group 1 belong to transpiration phenotype group A. All of them have an ABB genome constitution.

This experimental setup is useful and might be applied to other crops. Risk taking phenotypes investing in root growth might be rewarded, but climate change and especially variability is expected to lead to more unpredictability. We need to encourage farmers to spread their risk by planting different cultivars with their own conservative or risky physiology. So phenotyping and determining the risk physiology is of utmost importance. All lab models are limited by their experimental design and elimination of false positives through field validation trials is therefore essential. Nevertheless, lab models form an essential pre-breeding tool due to their specificity, control, and resource efficient high throughput. We conclude that lab models are very useful to study the biodiversity in great detail to identify traits that contribute to a better drought tolerance/avoidance. We encourage researchers investigating other crops to start to explore their collections.

## Data Availability

The datasets generated for this study are available on request to the corresponding author.

## Author Contributions

RS, TL, and SC supervised the experiments. SC designed the experiments. JvW, EK, and DE performed the experiments and analyzed the data. JvW and SC wrote the manuscript. All authors reviewed and approved the final manuscript.

## Conflict of Interest Statement

The authors declare that the research was conducted in the absence of any commercial or financial relationships that could be construed as a potential conflict of interest.

## Footnotes

1 https://www.crop-diversity.org/mgis/collection/01BEL084

2 http://www.cropontology.org/terms/CO_325/
