# Supplementary material for: Using Growth and Transpiration Phenotyping Under Controlled Conditions to Select Water Efficient Banana Genotypes
Source: Front Plant Sci. 2019 Mar 26;10:352. doi: 10.3389/fpls.2019.00352 (PMC6443892; doi:10.3389/fpls.2019.00352)
Supplement: Supplementary file 1 [file Data_Sheet_1.PDF]

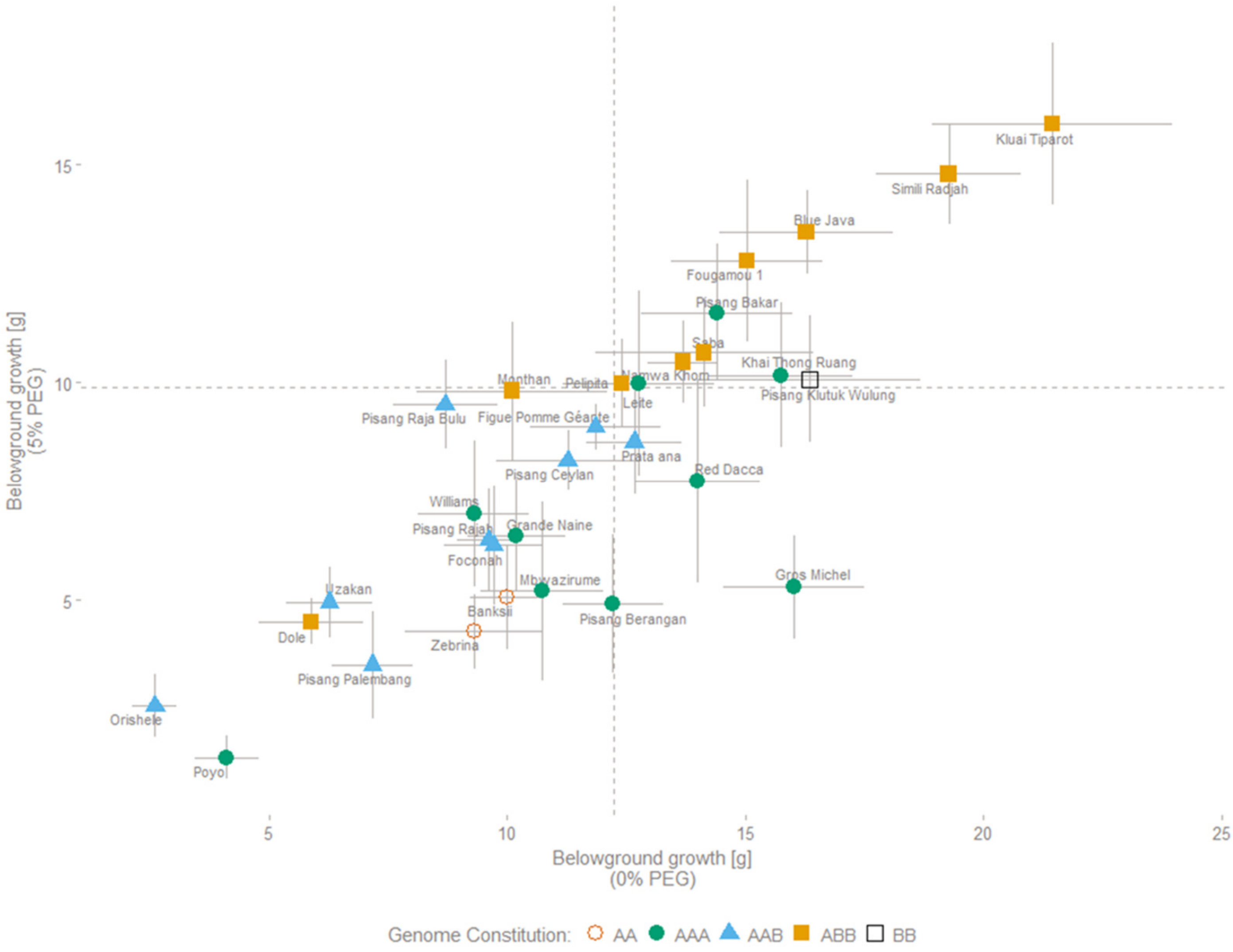

Supplementary figure 1: Belowground growth performance of 32 Musa biodiversity representatives. Performance is based on the belowground biomass production, in control (0% PEG) conditions (horizontal axis) and stressed (5% PEG) conditions (vertical axis). SE (n = 8) is indicated for control and stress for each cultivar. The cultivar genomic constitution (AA, AAA, AAB, ABB, or BB) is depicted by colors and shapes.
