# Supplementary material for: Using Growth and Transpiration Phenotyping Under Controlled Conditions to Select Water Efficient Banana Genotypes
Source: Front Plant Sci. 2019 Mar 26;10:352. doi: 10.3389/fpls.2019.00352 (PMC6443892; doi:10.3389/fpls.2019.00352)
Supplement: Supplementary file 2 [file Data_Sheet_2.PDF]

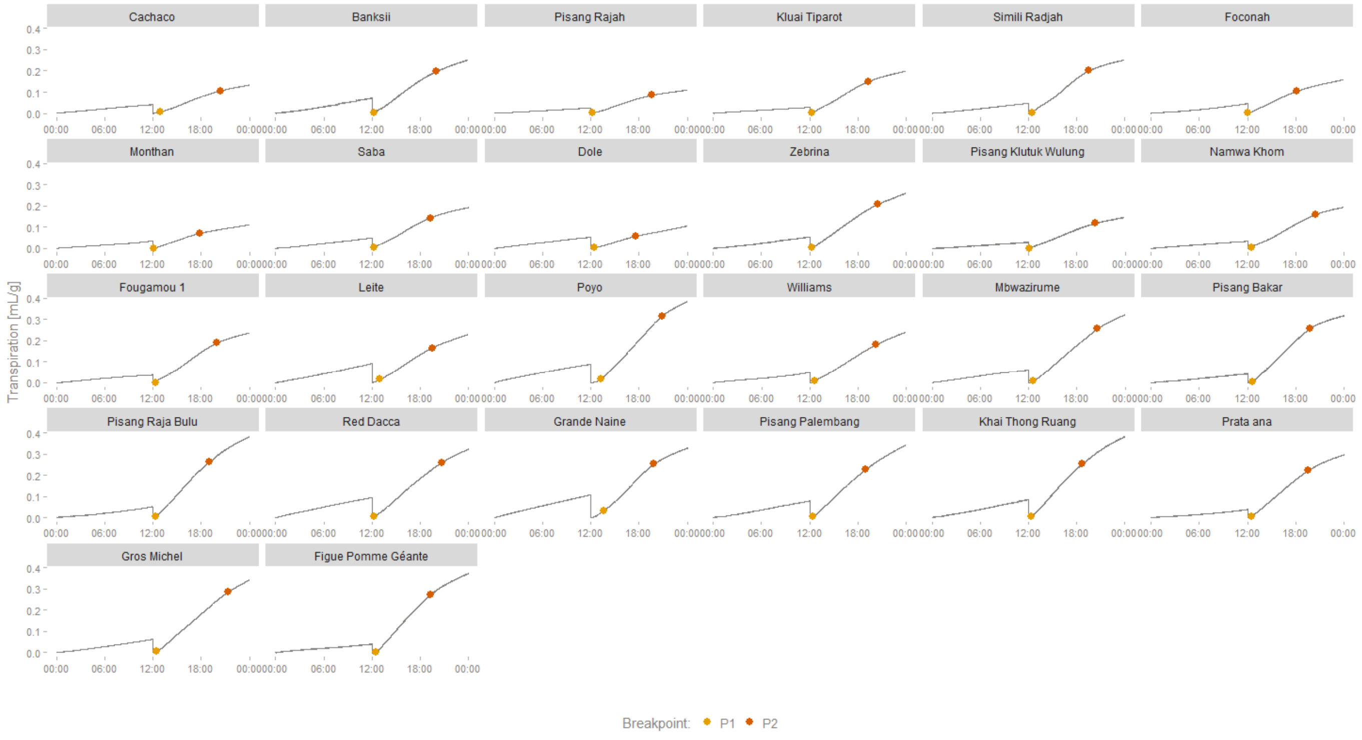

Supplementary figure 2: Per genotype the cumulative transpiration pattern of one sampled plant on one sample day is displayed, illustrating the variability between genotypes. The breakpoints (P1 and P2) from segmented regression analysis are indicated.
