# Supplementary material for: Using Growth and Transpiration Phenotyping Under Controlled Conditions to Select Water Efficient Banana Genotypes
Source: Front Plant Sci. 2019 Mar 26;10:352. doi: 10.3389/fpls.2019.00352 (PMC6443892; doi:10.3389/fpls.2019.00352)
Supplement: Supplementary file 3 [file Data_Sheet_3.PDF]

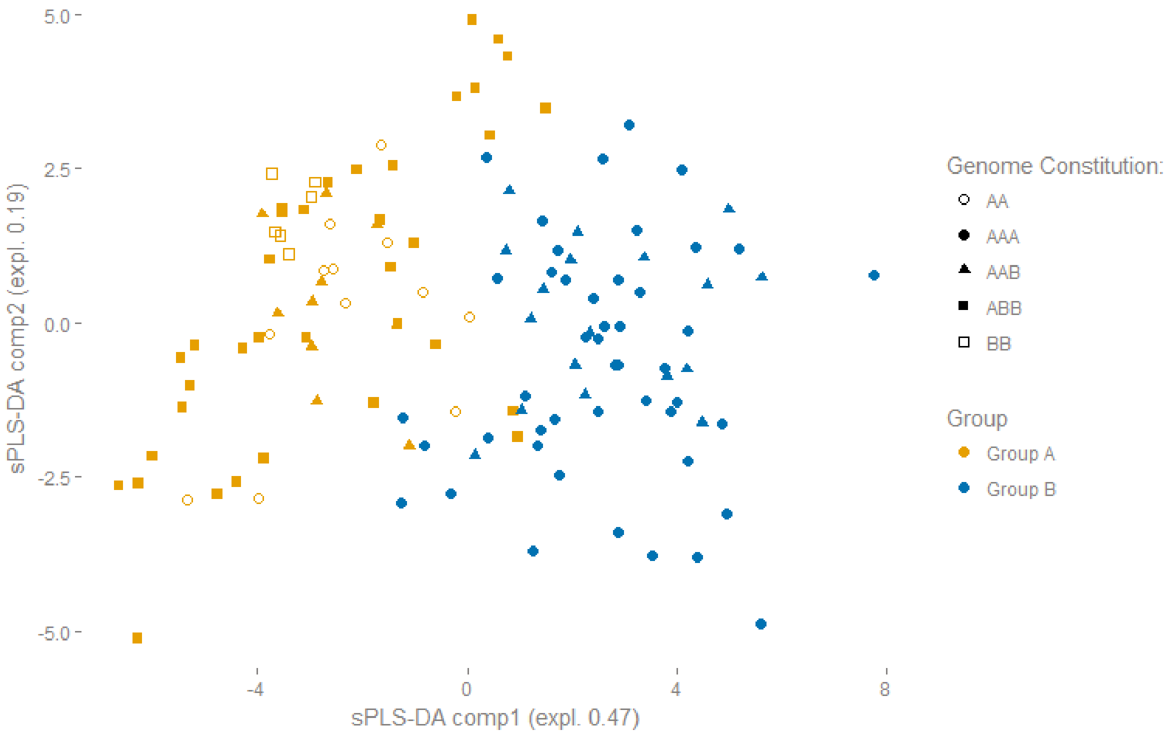

Supplementary figure 3: The transpiration dynamics group segregation can be visualized by sPLS-DA.
