# Supplementary material for: Using Growth and Transpiration Phenotyping Under Controlled Conditions to Select Water Efficient Banana Genotypes
Source: Front Plant Sci. 2019 Mar 26;10:352. doi: 10.3389/fpls.2019.00352 (PMC6443892; doi:10.3389/fpls.2019.00352)
Supplement: Supplementary file 4 [file Data_Sheet_4.pdf]

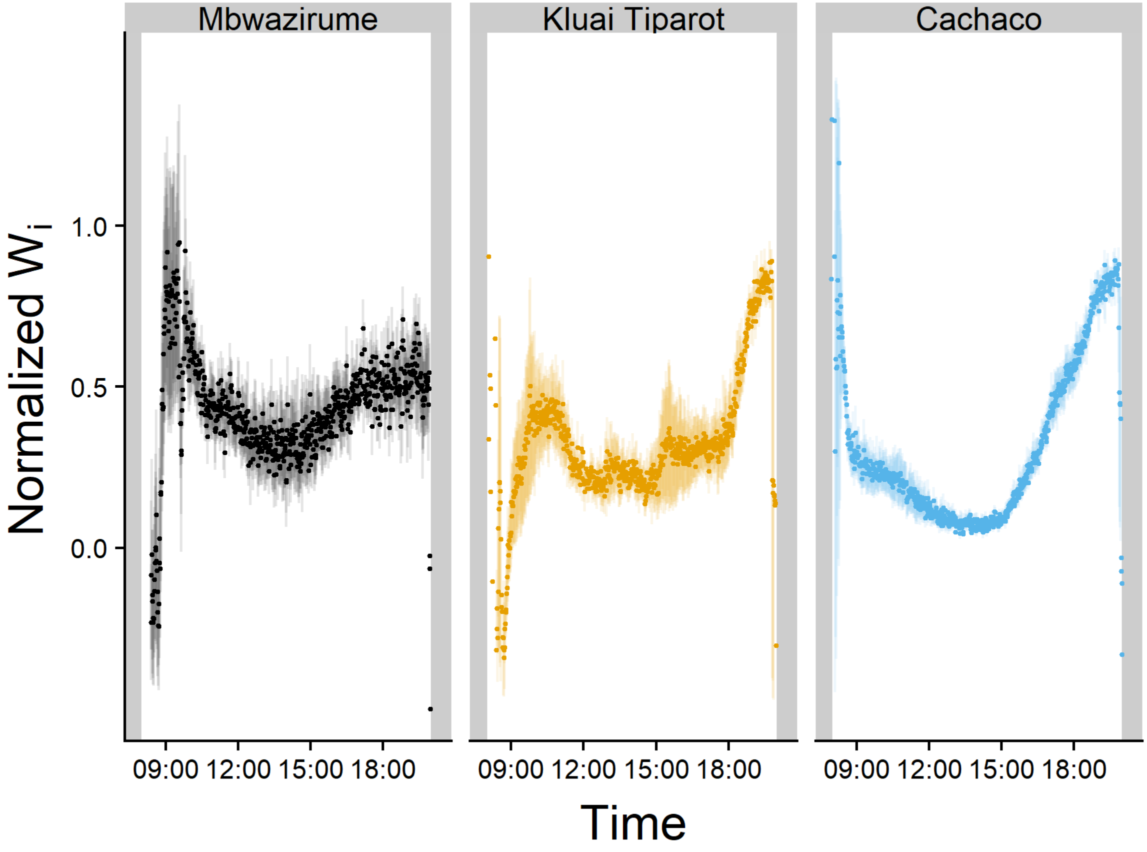

Supplementary figure 4: Normalized intrinsic water use efficiency profiles of three representative cultivars show an increase in WUE_i in the afternoon.
